# Synchronous GH- and prolactin-secreting pituitary adenomas

**DOI:** 10.1530/EDM-14-0052

**Published:** 2014-09-01

**Authors:** Maryam Rahman, Ignacio Jusué-Torres, Abdulrahman Alkabbani, Roberto Salvatori, Fausto J Rodríguez, Alfredo Quinones-Hinojosa

**Affiliations:** 1 Department of Neurosurgery, Johns Hopkins University, 600 North Wolfe Street, Phipps 1-111, Baltimore, Maryland, 21287, USA; 2 Division of Endocrinology, Department of Medicine, Johns Hopkins University, 600 North Wolfe Street, Phipps 1-111, Baltimore, Maryland, 21287, USA; 3 Department of Pathology, Johns Hopkins University600 North Wolfe Street, Phipps 1-111, Baltimore, Maryland, 21287, USA

## Abstract

Pituitary adenomas are usually solitary lesions. Rarely, patients may present with two distinct pituitary tumors. We report a case of synchronous secretory pituitary adenomas in a woman who initially presented with elevated prolactin levels. She was initially treated with cabergoline, but, after many years, she began developing symptoms consistent with acromegaly. Imaging revealed two distinct tumors within the pituitary gland. Endocrinological investigation confirmed acromegaly. At the time of surgery, two separate tumors were identified and resected. Pathological analysis demonstrated one tumor as a prolactinoma, and the other tumor as a GH-secreting adenoma. Postoperatively, her GH and IGF1 levels normalized, while the prolactin level remained slightly above normal. This case highlights that GH and prolactin level elevation is not always from co-secretion by the same adenoma.

**Learning points:**

- Synchronous pituitary adenomas represent <0.5% of pituitary tumors requiring surgery.
- In the setting of elevated GH and prolactin levels, one cannot assume that they are co-secreted by the same adenoma.
- A careful study of hormonal workup and pre-operative imaging is necessary for synchronous pituitary adenomas to assure resection of both tumors.

## Background

Pituitary adenomas are monoclonal cell-derived tumors, but can secrete more than one hormone. In the setting of simultaneous excessive growth hormone (GH) and prolactin secretion, the two hormones are most often secreted by the same adenoma. However, previous reports have described the rare occurrence of synchronous GH- and prolactin-secreting adenomas. We present a case of synchronous occurrence of a prolactinoma and somatotropinoma.

## Case presentation

A woman presented to an outside clinic at the age of 29 because of menstrual irregularities. She was found to have an elevated prolactin level (103 ng/ml, NV<23.3). According to outside reports, her other pituitary hormones including insulin-like growth factor 1 were normal. Imaging results from the time of diagnosis are not available, but she was diagnosed with a microadenoma. She was successfully treated with cabergoline with normalization of serum prolactin and regularization of menstrual periods. She was stable on this treatment for 11 years. At the age of 33, she was diagnosed with type 2 diabetes mellitus and was treated with oral agents. At the age of 40, her diabetes became more difficult to control. She underwent imaging that revealed a microadenoma and, while on cabergoline, her prolactin was 4.3 ng/ml. She also complained of fatigue and difficulty sleeping and was diagnosed with sleep apnea. Serum insulin-like growth factor 1 level was found to be elevated at 336 ng/ml (NV 62–205). She was referred to our clinic, and on questioning admitted to increase in ring and shoe size during the recent years. She denied a family history of pituitary, parathyroid, or pancreatic tumors.

## Investigation

**Figure 1:**
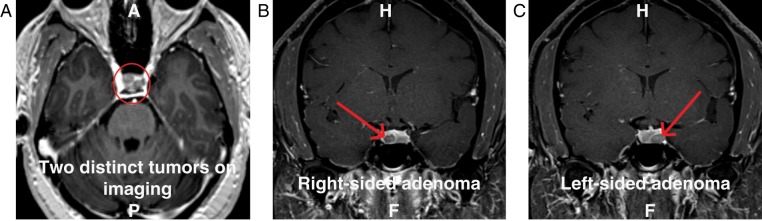
MRI demonstrating two distinct pituitary adenomas. (A) Axial MRI with contrast. (B) Coronal MRI with contrast. The arrow indicates the right-sided tumor. (C) Coronal MRI with contrast. The arrow indicates the left-sided tumor.

A glucose suppression test confirmed the diagnosis of acromegaly, with nadir GH level of 0.9 ng/ml after administration of 75 g of oral glucose. A brain magnetic resonance imaging was obtained and revealed two distinct tumors (Fig. 1). These lesions did not have any suprasellar extension and imaging characteristics were consistent with pituitary adenomas.

## Treatment

**Figure 2:**
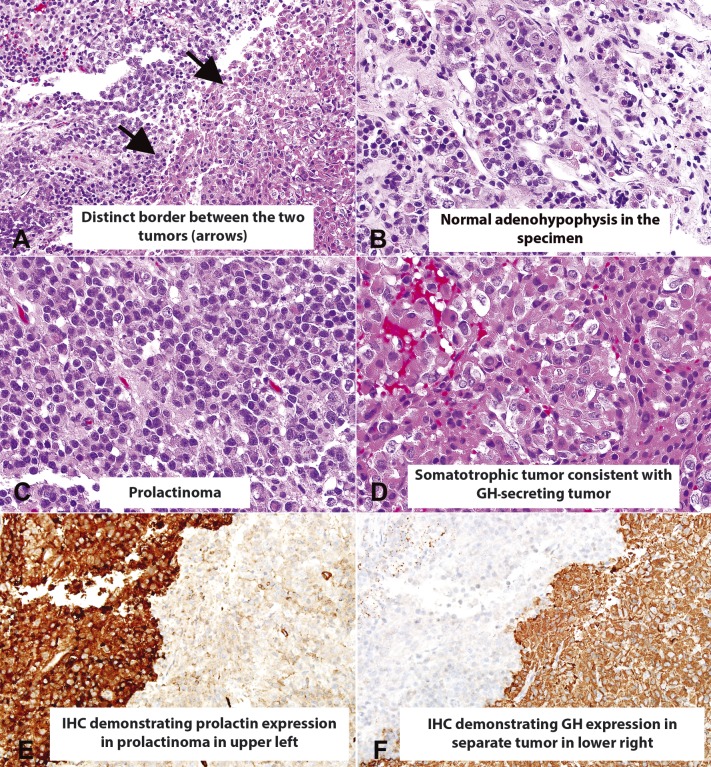
Histological sections of the left-sided excision demonstrated a sharp interface between the two adenomas (arrows), with an amphophilic prolactinoma (upper left) and an eosinophilic somatotrope (lower right) (A). A small fragment of adenohypophysis was also present (B). The prolactinoma was represented in the left-sided specimen (C), whereas the somatotrope showed prominent eosinophilia, consistent with a densely granulated subtype (D). Immunohistochemical stains confirmed strong, uniform prolactin staining in the prolactinoma (upper left) but not in the somatotrope (lower right) (E). Conversely, strong GH (GH1) expression was present in the somatotrope (lower right) but not in the prolactinoma (upper left) (F).

The patient underwent endoscopic transsphenoidal surgery with use of stereotaxis. Two distinct tumors were identified and were resected as two separate specimens. Pathology confirmed two distinct tumors (Fig. 2). One of these tumors consisted only of somatotropic cells and the other was a completely lactrotropic tumor.

## Outcome and follow-up

The patient had an uneventful post-operative course and her GH and insulin-like growth factor 1 normalized. The GH level was <0.1 ng/ml at 1 month after surgery, and the insulin-like growth factor 1 level was 221 and 226 ng/ml (NV 52–328) at one and six months after surgery respectively. Conversely, her prolactin level remained abnormal at 28.0 (one month post-operatively) and 29.3 (3 months post-operatively) and 42.6 ng/ml (6 months post-operatively) (NV<23.3), showing that the prolactin-secreting tumor was not completely resected. Cabergoline was re-started six months after surgery.

## Discussion

Pituitary gland tumors are present in up to 20% of the population and a third of these are clinically significant (1). The presence of two distinct adenomas has been reported in <0.5% of patients undergoing surgery for pituitary tumors (2) (3). Pituitary adenomas are thought to be monoclonal based on X-chromosome inactivation patterns (4).

Approximately 25% of GH-secreting adenomas co-secrete prolactin. These include dimorphous adenomas composed of GH and prolactin cells, monomorphous mammosomatotrope adenomas (which produce both GH and prolactin), and rarely primitive and often aggressive acidophil stem-cell adenomas (5). However, previous reports have also described rare synchronous GH- and prolactin-secreting adenomas (6) (7). In some of these cases, the patients had familial MEN1 syndrome (7). Therefore, a careful family history should be collected in these cases. However, these synchronous adenomas may also occur outside familial disease, as in our patient's case who did not have a family history consistent with MEN1 or familial isolated pituitary adenoma syndrome.

This case highlights that, in the setting of simultaneous excessive GH and prolactin secretion, one cannot assume that the two hormones are secreted by the same adenoma and that, when both GH and prolactin are abnormal, a very careful pre-operative review of magnetic resonance imaging must be carried out to identify the possible presence of two distinct adenomas.
